# Proteomics Standards Initiative’s ProForma 2.0: Unifying the Encoding of Proteoforms and Peptidoforms

**DOI:** 10.1021/acs.jproteome.1c00771

**Published:** 2022-03-15

**Authors:** Richard D. LeDuc, Eric W. Deutsch, Pierre-Alain Binz, Ryan T. Fellers, Anthony J. Cesnik, Joshua A. Klein, Tim Van Den Bossche, Ralf Gabriels, Arshika Yalavarthi, Yasset Perez-Riverol, Jeremy Carver, Wout Bittremieux, Shin Kawano, Benjamin Pullman, Nuno Bandeira, Neil L. Kelleher, Paul M. Thomas, Juan Antonio Vizcaíno

**Affiliations:** 1 National Resource for Translational and Developmental Proteomics, Northwestern University, Evanston, IL, 60611, USA; 3 Institute for Systems Biology, Seattle WA 98109, USA; 4 Clinical Chemistry Service, Lausanne University Hospital, 1011 Lausanne, Switzerland, 1007; 5 Department of Genetics, Stanford University, Stanford, CA 94305, USA; 6 Chan Zuckerberg Biohub, 499 Illinois St, San Francisco, CA 94158, USA; 7 SciLifeLab, School of Engineering Sciences in Chemistry Biotechnology and Health, KTH − Royal Institute of Technology, SE-171 21 Solna, Stockholm, Sweden, 113 51; 8 Program for Bioinformatics, Boston University, Boston, MA 02215, USA; 9 VIB − UGent Center for Medical Biotechnology, VIB, Technologiepark 75 - FSVM II, 9052 Ghent, Belgium; 10 Department of Biomolecular Medicine, Faculty of Medicine and Health Sciences, Ghent University, 9000 Ghent, Belgium; 11 European Molecular Biology Laboratory, EMBL-European Bioinformatics Institute (EMBL-EBI), Hinxton, Cambridge, CB10 1SD, United Kingdom; 12 Center for Computational Mass Spectrometry, University of California, San Diego (UCSD), La Jolla, CA 92093, USA; 13 Dept. Computer Science and Engineering, University of California, San Diego (UCSD), La Jolla, CA 92093, USA; 14 Skaggs School of Pharmacy and Pharmaceutical Sciences, University of California, San Diego (UCSD), La Jolla, CA 92093, USA; 15 Toyama University of International Studies, Toyama. 930-1292 Toyama, Higashikuromaki, 6 5-1, Japan; 16 Database Center for Life Science, Joint Support-Center for Data Science Research, Research Organization of Information and Systems, Kashiwa, Chiba 277-0871, Japan

**Keywords:** ProForma, proteoform, peptidoform, top-down proteomics, file formats, data standards, mass spectrometry, FAIR

## Abstract

It is important for the proteomics community to have a standardized manner to represent all possible variations of a protein or peptide primary sequence, including natural, chemically-induced and artifactual modifications. The Human Proteome Organization (HUPO) Proteomics Standards Initiative (PSI) in collaboration with several members of the Consortium for Top-Down Proteomics (CTDP) has developed a standard notation called ProForma 2.0, which is a substantial extension of the original ProForma notation developed by the CTDP. ProForma 2.0 aims to unify the representation of proteoforms and peptidoforms.

ProForma 2.0 supports use cases needed for bottom-up and middle-/top-down proteomics approaches and allows the encoding of highly modified proteins and peptides using a human-and machine-readable string. ProForma 2.0 can be used to represent protein modifications in a specified or ambiguous location, designated by mass shifts, chemical formulas, or controlled vocabulary terms, including cross-links (natural and chemical), and atomic isotopes. Notational conventions are based on public controlled vocabularies and ontologies. The most up-to-date full specification document and information about software implementations are available at http://psidev.info/proforma.

## Introduction

Protein and peptide sequences are usually represented by a string of amino acids using the well-known one-letter code that was first introduced by the International Union of Pure and Applied Chemistry (IUPAC) in 1972.^1^ The linear arrangement of the amino acids is customarily written from the *N*-terminus to the *C*-terminus. However, there is no clear consensus about how to represent amino acid modifications, which can be natural [e.g., biologically-relevant post-translational modifications (PTMs)], chemically-induced (including, for example, reduction/alkylation and addition of tags for quantitative analysis) or artifactual as a consequence of sample preparation (such as oxidation and deamidation).

The terms “proteoform”^2^ and “peptidoform,”^3^ are used for the specific “form” or “entity” of a given protein or peptide that results from the combination of the amino acid sequence and modification(s) at specific amino acid positions. Multiple proteoforms can be derived from the same gene. For example, if a protein has two sites that can potentially be phosphorylated, there are four possible proteoforms: the unmodified form represented by the primary sequence, and the forms with phosphorylation on the first site, the second site, and both sites. Each of these are distinct proteoforms, but only the first proteoform, the unmodified variant, can be written using the IUPAC notation. In the absence of a recognized standard notation, there is no consistency in the way modified proteins and peptides are designated. This can not only lead to confusion in scientific publications and presentations, but it is also a major dilemma for developers of proteomics software and resources to decide what notation(s) to use for data input and output. This is applicable to widely-used protein-centric database resources such as UniProtKB (UniProt Knowledge-Base),^4^ ProteomeXchange proteomics resources,^5^ the Protein Data Bank (PDB),^6^ Reactome^7^ and IntAct,^8^ among many others. This has led to the development of multiple different notational formats by various groups.

In order to make peptide and protein data more *findable*, *accessible*, *interoperable* and *reusable* (FAIR),^9^ there needs to be a single IUPAC-compatible notational standard to encode modified protein and peptide sequences. In 2018, the Consortium for Top-Down Proteomics (CTDP) introduced the ProForma notation,^10^ which answered the immediate needs of the Consortium by creating a standardized method for designating a proteoform. It contained seven rules to denote both the primary structure of a proteoform and most of the commonly-observed PTMs and artifactual modifications, using nomenclature from five ontologies and controlled vocabularies (CVs). In general, CVs are minimally structured lists of terms and definitions, while ontologies encode the full hierarchical relationship structure among the terms^11^.

However, this notational system was not sufficient to meet the needs of the broader proteomics community and protein data resources because some important use cases were not supported. In particular, the first ProForma version did not address issues such as ambiguity in either the order of the amino acid sequence or modification site localization, and did not support cross-links (natural or chemically-induced), among many others. For proteoform and peptidoform designations to be FAIR across the broader array of protein science data resources, these and numerous other notational issues needed to be addressed. Ideally, the same notational system should be usable for both bottom-up and middle-/top-down applications.

The Proteomics Standards Initiative (PSI) of the Human Proteome Organization (HUPO) develops and ratifies community-based data standards and CVs for the field of proteomics,^12^ including mzML,^13^ mzIdentML,^14^ mzTab,^15^ PSI-MOD,^16^ PEFF (PSI Extended FASTA Format)^17^ and more recently, the Universal Spectrum Identifier (USI)^18^ and the sample metadata standard MAGE-TAB-Proteomics.^19^ Each of these standards has been subjected to the PSI Document Process^20^ which mandates three levels of review that must be completed before a proposed standard is ratified. In order to address the use cases needed for bottom-up and middle-/top-down approaches, members of the CTDP and HUPO-PSI worked together and devised an extended ProForma notation designed to meet the current and future needs for protein sequence data. In this article, we present an overview of the ProForma 2.0 notation, a brief description of its most salient features, and some example applications.

## Methods

### Development of ProForma 2.0

The development of ProForma 2.0 started in 2019. Since then, it was an open process via conference calls in addition to discussions at the annual PSI meetings and smaller workshops. The ProForma 2.0 specification document was submitted to the PSI Document Process for review, during which time external reviewers provided their feedback. The document was also made available for comments by the public, enabling broad input on the specifications. The final version of the ProForma 2.0 specification document is provided as Supplementary Document 1. Potential corrections to the document, up-to-date information on software implementations, and information on future versions of ProForma are available at http://psidev.info/proforma.

The main requirements considered during the development of the standard notation were:

1. It must be a string of characters that is human-readable, i.e. it should be suitable for display in a written document or in a presentation.
2. It must be unambiguously parsable by software (i.e., machine-parsable).
3. It must be able to support the encoding of amino acid sequences and their modifications (including natural, chemically-induced and artifactual).
4. It must be able to support the main use cases needed by the proteomics community as a whole, including bottom-up (focused on peptides/peptidoforms) and middle-/top-down (focused on proteins/proteoforms) applications.
5. It must be flexible enough to accommodate different styles of notations that are currently in common use.
6. It must be compatible with other existing PSI file formats.
7. It must be able to accommodate ambiguity in the position of a modified site.
8. It must be able to evolve so that new use cases can be added in the future.

Requirements 1 − 3 were included in the original ProForma 1.0 notation.^10^ The essence of the fourth requirement was in the ProForma 1.0 notation, but the current version now includes support for bottom-up proteomics-specific entities, i.e. for peptidoforms, whereas the original exclusively defined the way to designate whole proteoform sequences. Requirements 4 − 8 are new in ProForma 2.0.

An essential requirement of ProForma 2.0 is that it should be able to represent peptidoforms and proteoforms in a consistent and reproducible way, taking into consideration the different strategies for designating protein modifications. Moreover, it must be able to be used jointly with USIs^18^ to represent peptide spectrum matches (PSMs) and proteoform spectrum matches (PrSMs).

## Results

### Data Format Description

**Figure 1:**
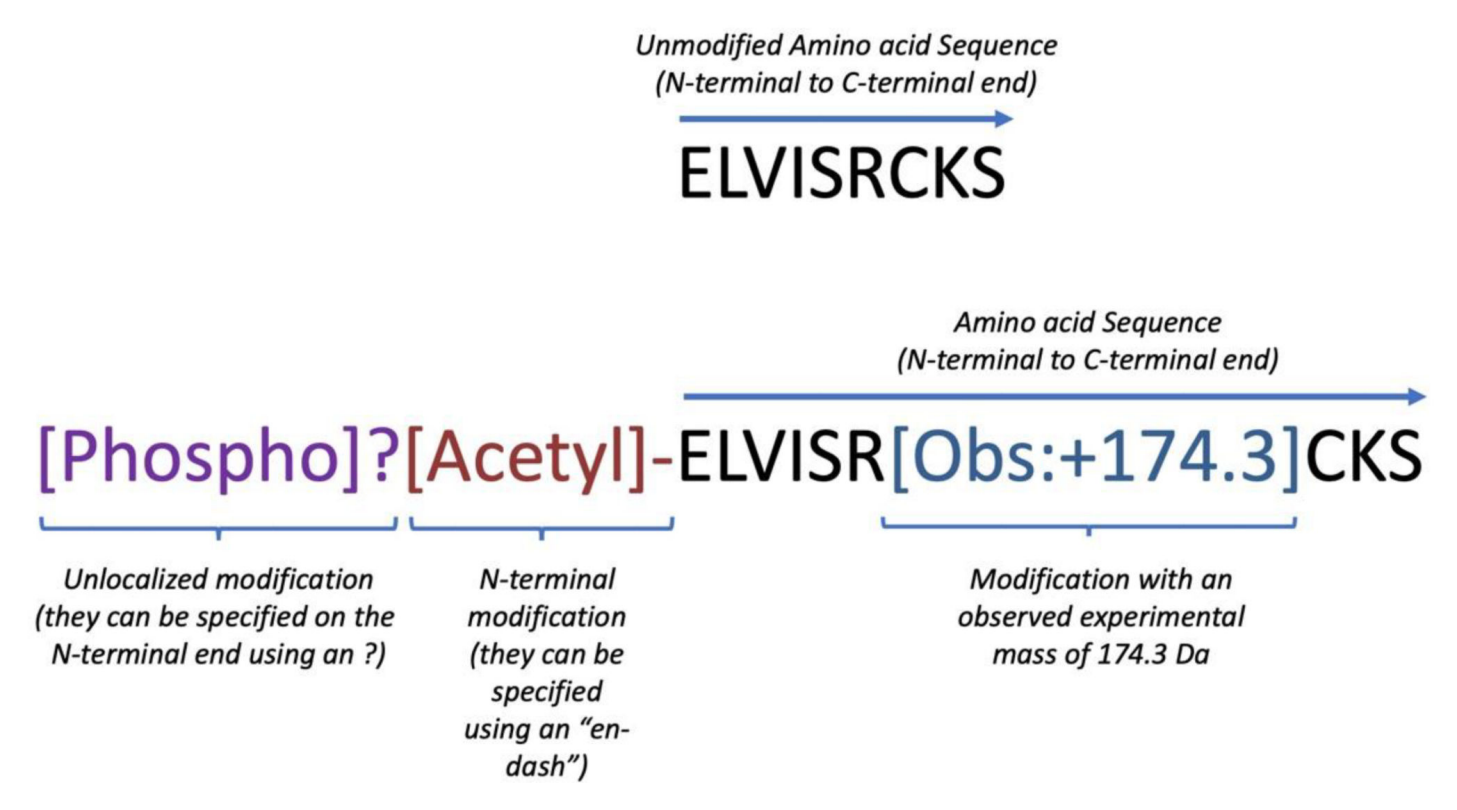
Representation of the same *N*-terminal segment (sharing the same amino acid sequence) of two hypothetical proteoforms using ProForma 2.0: the unmodified proteoform (top part of the figure) and one containing different protein modifications (lower part of the figure). The text coloration is only included here to improve clarity. The purple tag encodes the existence of an unlocalized phosphorylation event somewhere on the proteoform. The keyword “Phospho” is from Unimod and can be used without additional clarification. The brown tag is a reference to an *N*-terminal modification using the term ”Acetyl” from Unimod. A 174.3-Da mass shift on the arginine is also indicated.

Here we provide a brief overview with examples of the main features of ProForma 2.0, while the full ProForma 2.0 specification document, as ratified by the PSI, provides exhaustive details on all aspects of the data format. ProForma 2.0 provides a standardized set of rules for describing the location and nature of all mass modifications on a proteoform or peptidoform. An example is shown in Figure 1. Using ProForma 2.0, there is a string of characters that linearly represents the peptidoform/proteoform primary structure, with allowance for some level of ambiguity, and the possibility to link peptide chains together, such as by cross-linking. ProForma 2.0 is not intended to represent secondary or higher-order structures. ProForma 2.0 can also be used to represent the molecular interpretation of a tandem mass spectrum. It should be noted that ProForma 2.0 is designed to describe a single, specific peptidoform or proteoform and not a collection of protein sequences or a listing of all potential mass modifications that may be found on them (i.e., a protein sequence search database). Other file formats such as PEFF^17^ are better suited for this purpose.

When using the ProForma 2.0 notation for peptidoforms and proteoforms, amino acids are shown as is customary from left to right, *N*- to *C*-terminus, using IUPAC single letter identifiers. Modifications of this core set of amino acids are designated by a coded string of characters enclosed in square brackets after the letter of the modified residue. The modification string is represented by CV or ontology terms. The supported CVs/ontologies in ProForma 2.0 are PSI-MOD,^16^ Unimod,^21^ RESID,^22^ XL-MOD (cross-linking; https://github.com/HUPO-PSI/xlmod-CV) and the Glycan Naming Ontology (GNO; glycans; https://www.ebi.ac.uk/ols/ontologies/gno).

ProForma 2.0 is case insensitive. This means that the notation is agnostic with regard to the use of uppercase or lowercase characters. However, different CVs and/or ontologies generally have their own specific policies for capitalization and representation of terms. It is, therefore, recommended that the capitalization specifications for each supported CV/ontology be used. It is also important to highlight that line breaks must not be used. There is currently no limit in maximum length since ProForma 2.0 can be used to represent both peptidoforms and proteoforms. Additionally, non-ASCII (American Standard Code for Information Interchange) characters are allowed since they may be included in the supported terms in the different CVs/ontologies.

A comparison of the features of ProForma 1.0 (finished in 2018) and 2.0 is shown in Table 1. At least 18 features were either added or expanded. Examples of ProForma 2.0 notations are provided in Table 2, along with the section number in the specification document (Supplementary Document 1) that contains the detailed description of each feature. Note that custom user-specific information may be added to ProForma 2.0 entities by means of using “Information tags.” Additionally, in the 2.0 version, the use of “Information tags” is the only mechanism to add metadata for a ProForma entity.

### Levels of Compliance

It is important to highlight that software that implements the ProForma 2.0 notation may not support all aspects of the specification. For example, a standard proteomics search engine that outputs the ProForma notation does not have to support the cross-linking part of the notation. We have, therefore, defined five levels of ProForma 2.0 compliance (listed below) in order to make adoption easier. Details can be found in the specification document (Supplementary Document 1, Appendix I).

Base Level (“Base-ProForma Compliant”).

Level 2 (“Level 2-ProForma compliant”).

Top-Down Extensions (Level 2-ProForma + top-down compliant).

Cross-Linking Extensions (Level 2-ProForma + cross-linking compliant).

Glycan Extensions (Level 2-ProForma + glycans compliant).

More than one of the extensions listed above (top-down, cross-linking and glycan) could be supported by the same software.

### Software Implementations

ProForma 2.0 has already been implemented in some existing software. The CTDP has established an initial proteoform registry where experimentally verified proteoforms are assigned a unique PFR (ProteoForm Record) identifier (http://www.proteoform.org/api).^23^ This identifier system is essential for enhancing interoperability between tools and databases that include proteoform data. The registry is based on an API (Application Programming Interface) that accepts ProForma 2.0 sequences, compares them to known proteoforms already stored in the registry, and returns a new PFR identifier, if the proteoform is new to the system. However, if the proteoform is already stored in the registry, a PFR identifier generated previously is returned. Then, ProForma 2.0 is needed as an input to the registry so that PFR identifiers can be provided.

There are currently four implementations of parsers and writers for ProForma 2.0, including the following:

1. A .NET version, as part of the Top-Down Software Development Kit (SDK) (https://github.com/topdownproteomics/sdk). This includes a lexer/parser with some additional proteoform validation functionality.
2. A Java port of the .NET reader and writer (https://github.com/NRTDP/proforma-java).
3. A Python version of a parser and writer, which is now part of the Pyteomics ^24^ framework (https://github.com/levitsky/pyteomics). Additional documentation is available here (https://pyteomics.readthedocs.io/en/latest/api/proforma.html).
4. The spectrum_utils Python package ^25^ includes a parser using a formal grammar to convert ProForma strings into abstract syntax trees (https://github.com/bittremieux/spectrum_utils/).

ProForma strings are also an optional part of the recently developed USI standard for representation of PSMs (see some examples at http://proteomecentral.proteomexchange.org/usi/). We expect that adoption of ProForma will increase broadly in the field, stimulated by its inclusion in widely-used bioinformatics resources such as those created by the CTDP, ProteomeXchange^5^ and UniProtKB,^4^ among others.

## Discussion and Conclusions

ProForma 2.0 is a standard notation that is capable of supporting the needs of both the bottom-up and the top-down proteomics communities. Since peptidoforms and proteoforms are easily encoded in the ProForma 2.0 notation, it simplifies comparing the results of different search engines. This will greatly facilitate reuse of experimental data. We also anticipate that the ProForma 2.0 notation will expedite integration of bottom-up and middle-/top-down data, which is an active field of research.^26, 27^ Moreover, the notation can be used as an input for the first version of the Proteoform Registry, which generates of unambiguous PFR identifiers for proteoform entities. Use of PFR identifiers is key to facilitate proteoform data interoperability between multiple tools and protein databases.

Proforma 2.0 has been developed as a joint effort between the PSI and the CTDP and will be actively maintained. Both organizations expect that this version 2.0 will not change for an extended period of time since it addresses most of the relevant use cases at the time of writing. However, additional use cases have already been envisioned and documented in the specification document (see Section 5, “Pending Issues - Future developments,” in Supplementary Document 1). We expect that these extra features can be addressed in future versions, after the community has gained experience with the more common use cases included in version 2.0. The current list of known open issues includes: representation of cyclic peptides, representation of more complex scenarios where there is ambiguity in the localization of different glycans attached to the same amino acid sequence, support for rare amino acids which are not assigned to an accepted one-letter code, support the use of average masses in the notation, lipid modifications, support for molecular formulas, overlapping ranges of possible protein modification localizations, ambiguous cross-linker modification positions, representation of the distribution of different isotopes in the sequence, and the representation of sequences coming from non-MS-based proteomics approaches (e.g. peptide nanopores and Edman-based sequencing).

PSI standards are developed via an open process in which all interested individuals and groups are encouraged to participate. ProForma 2.0 has been developed by contributors from both the top-down and bottom-up proteomics subfields. This fusion provides the community with a standard that supports a diverse array of use cases and creates the potential for a substantially higher degree of software tool interoperability within the field than in the past. Although standards that are cooperatively developed inevitably take longer to complete than formats proposed by a single group, the resulting standards are more broadly applicable to many more use cases than those from independent initiatives. Broad participation is, therefore, essential for successful generation of future standards for the proteomics community. See https://www.topdownproteomics.org/ to become involved in the top-down proteomics activities of the CTDP and https://psidev.info/ for information about how to contribute to the PSI.

## Supplementary Material

Supporting Information

## Figures and Tables

**Table 1:** Comparison of the supported features of ProForma 1.0 and 2.0.

Feature	ProForma 1.0	ProForma 2.0
Protein modifications designated by CV/ontology names and accession numbers	✓	✓
Representation of glycan composition	✓	✓
*N*-terminal and *C*-terminal modifications	✓	✓
Delta mass notation for modifications	✓	✓
Information tag	✓	✓
Joint representation of experimental data and interpretation	✓	✓
NEW Support for elemental formulas	Limited	✓
NEW Representation of isotopes	Limited	✓
NEW Cross-link notation	X	✓
NEW Representation of inter-chain cross-links	X	✓
NEW Representation of disulfide linkages	X	✓
NEW Representation of glycans with GNO ontology as CV	X	✓
NEW Specifying a gap of known mass	X	✓
NEW Labile modifications	X	✓
NEW Unknown modification position	X	✓
NEW Possible set of modification positions	X	✓
NEW Representing ranges of positions for the modifications	X	✓
NEW Modification position preference and localization scores	X	✓
NEW Scoring for ranges of positions for a modification	X	✓
NEW Fixed protein modifications	X	✓
NEW Ambiguity in the order of amino acid sequences	X	✓
NEW Representation of ion charges and more than one peptidoform per spectrum	X	✓
NEW Representation of branched peptides	X	✓
NEW Representation of ambiguity in the order of the amino acid sequence	X	✓

**Table 2:** Examples of ProForma 2.0 notations demonstrating the various features of the specification. For each feature listed in the first column, there is a representative example in the second column showing the encoding. The “Section” column provides the location in the PSI specification document where the feature is explained in detail (Supplementary Document 1).

Feature	Example	Section
CV/ontology modification names	EM[Oxidation]EVEES[Phospho]PEK	4.2.1
CV/ontology protein modification accession numbers	EM[MOD:00719]EVEES[MOD:00046]PEK	4.2.2
Cross-link within the same peptide	EMEVTK[XLMOD:02001#XL1]SESPEK[#XL1]	4.2.3.1
Inter-chain cross-links	SEK[XLMOD:02001#XL1]UENCE//EMEVTK[#XL1]SESPEK	4.2.3.2
Disulfide linkages	EVTSEKC[MOD:00034#XL1]LEMSC[#XL1]EFD	4.2.3.3
Branched peptides	ETFGD[MOD:00093#BRANCH]//R[#BRANCH]ATER	4.2.4
Glycans using the GNO ontology as CV	NEEYN[GNO:G59626AS]K	4.2.5
Delta mass notation for modifications	EM[+15.9949]EVEES[+79.9663]PEK	4.2.6
Specifying a gap of known mass	RTAAX[+367.0537]WT	4.2.7
Support for elemental formulas	SEQUEN[Formula:C12H20O2]CE	4.2.8
Glycan composition	SEQUEN[Glycan:HexNAc1Hex2]CE	4.2.9
*N*-terminal and *C*-terminal modifications	[iTRAQ4plex]-EMEVNESPEK	4.3.1
Labile modifications	{Glycan:Hex}EMEVNESPEK	4.3.2
Unknown modification position	[Phospho]?EMEVTSESPEK	4.4.1
Possible set of modification positions	EMEVT[#g1]S[#g1]ES[Phospho#g1]PEK	4.4.2
Ranges of positions for the modifications	PROT(ESFRMS)[+19.0523]ISK	4.4.3
Modification position preference and localization scores	EMEVT[#g1(0.01)]S[#g1(0.09)]ES[Phospho#g1(0.90)]PEK	4.4.4
Scoring for ranges of positions for a modification	PROT(ESFRMS)[+19.0523#g1(0.01)]ISK[#g1(0.99)]	4.4.5
Isotopes	<13C>ATPEILTVNSIGQLK	4.6.1
Fixed protein modifications	<[MOD:01090]@C>ATPEILTCNSIGCLK	4.6.2
Ambiguity in the order of the amino acid sequence	(?DQ)NGTWEMESNENFEGYMK	4.7
Information tag	ELVIS[Phospho|INFO:newly discovered]K	4.8
Joint representation of experimental data and interpretation	ELVIS[Phospho|Obs:+79.978]K	4.9
Representation of ion charges	EMEVEESPEK/2	7.1
Multiple peptidoforms assigned to chimeric spectra	EMEVEESPEK/2+ELVISLIVER/3	7.1

## Abbreviations

### Abbreviations

API: Application Programming Interface
ASCII: American Standard Code for Information Interchange
CTDP: Consortium for Top-Down Proteomics
CV: Controlled Vocabulary
FAIR: Findable, Accessible, Interoperable and Reusable
GNO: Glycan Naming Ontology
HUPO: Human Proteome Organization
IUPAC: International Union of Pure and Applied Chemistry
MS: Mass Spectrometry
PDB: Protein Data Bank
PEFF: PSI Extended FASTA Format
PFR: Proteoform Record
PSI: Proteomics Standards Initiative
PrSM: Proteoform Spectrum Match
PSM: Peptide-Spectrum Match
PTM: Post-Translational Modification
SDK: Software Development Kit
UniProtKB: UniProt Knowledge-Base
USI: Universal Spectrum Identifier
